# Evaluating a Board Game Designed to Promote Young Children’s Delay of Gratification

**DOI:** 10.3389/fpsyg.2020.581025

**Published:** 2020-11-11

**Authors:** Stephanie Anzman-Frasca, Anita Singh, Derek Curry, Sara Tauriello, Leonard H. Epstein, Myles S. Faith, Kaley Reardon, Dave Pape

**Affiliations:** ^1^Department of Pediatrics, Jacobs School of Medicine and Biomedical Sciences, University at Buffalo, Buffalo, NY, United States; ^2^Center for Ingestive Behavior Research, University at Buffalo, Buffalo, NY, United States; ^3^Cedars-Sinai Samuel Oschin Comprehensive Cancer Institute, Cedars-Sinai Medical Center, Los Angeles, CA, United States; ^4^College of Arts, Media and Design, Northeastern University, Boston, MA, United States; ^5^Department of Counseling, School, and Educational Psychology, Graduate School of Education, University at Buffalo, Buffalo, NY, United States; ^6^Department of Media Study, College of Arts and Sciences, University at Buffalo, Buffalo, NY, United States

**Keywords:** delay of gratification, children, board game, game-based learning, self-regulation

## Abstract

**Objective:**

Delay of gratification, or the extent to which one can resist the temptation of an immediate reward and wait for a larger reward later, is a self-regulatory skill that predicts positive outcomes. The aim of this research was to conduct initial tests of the effects of a board game designed to increase children’s delay of gratification via two experimental studies.

**Methods:**

Preschool children were randomized to play the study game or a control game. In Study 1, there were 48 children in the analytic sample, with a mean age of 4.81 ± 0.55 years; Study 2 included 50 children (*M* = 4.02 ± 0.76 years). Delay of gratification was assessed during the study game, as well as before and after game play sessions using the Marshmallow Test.

**Results:**

In both studies, the intervention group’s likelihood of delaying gratification during the study game increased across game-play sessions (*p* < 0.05). In Study 1, the intervention group also increased wait times during the Marshmallow Test versus controls (*p* = 0.047). In Study 2, there was no effect on Marshmallow Test wait times.

**Conclusion:**

Results provide some initial evidence supporting potential efficacy of a board game designed to increase delay of gratification. Future research can clarify: (1) which components of game play (if any) are linked with broader changes in delay of gratification, (2) impacts of this intervention in more diverse samples, and (3) whether experimental manipulation of delay of gratification affects outcomes like achievement and weight, which have been linked to this skill in observational studies.

## Introduction

Delay of gratification (DG) is the extent to which one can resist the temptation of an immediate reward and wait for a larger, later reward. Preschoolers’ DG predicts positive outcomes across developmental domains, including better academic performance, greater social competence, and healthier weight outcomes in adolescence and beyond (Mischel et al., 1988, 1989; Francis and Susman, 2009; Casey et al., 2011; Schlam et al., 2013). Developing innovative, low-cost ways to promote DG during early childhood offers the potential to equip children with a useful skill for navigating the modern environment.

Delay of gratification is a self-regulatory skill (Duckworth et al., 2013). It involves the use of inhibitory control: restraining a dominant, or desired, response and executing a subdominant response instead (Rothbart et al., 2004). Many developmental tasks involve inhibitory control. For example, in school contexts, children may be asked to complete a learning exercise that involves remembering directions, taking turns, and staying on task (McClelland and Cameron, 2012). Inhibitory control is needed to resist the temptation of distractions and complete such activities successfully. Consistent with this idea, there is evidence from traditional curriculum-based preschool interventions that effects on school readiness skills were mediated by improvements in self-regulation (Bierman et al., 2008; Raver et al., 2011).

Children from lower socioeconomic status (SES) households are less likely to delay gratification compared to their higher-SES counterparts, and these differences may contribute to disparities like the achievement gap (Evans and Rosenbaum, 2008). There is evidence supporting the idea that these differences reflect contextually appropriate responses to less-predictable environments, rather than deficits (Sturge-Apple et al., 2016; Pepper and Nettle, 2017). Providing experiences in which the act of delaying gratification pays off may counter these effects, at least in those contexts that are shown to be predictable (Michaelson and Munakata, 2020).

While effective curriculum-based programs for promoting broader self-regulation skills exist, there is a dearth of research testing interventions with the potential to enhance DG specifically, as noted in several recent publications (Watts et al., 2018; Haimovitz et al., 2019; Falk et al., 2020). Such investigations can help to elucidate whether the potential of interventions to promote self-regulation extends to DG specifically, and if this is the case, these studies can also clarify whether observed links between DG and subsequent outcomes are causal.

Game-based learning offers a promising and innovative intervention approach. Previously researchers have used circle-time games in classroom settings to increase self-regulation among preschoolers from low-income households (Tominey and McClelland, 2011; Schmitt et al., 2015). In these studies, a variety of games were administered during circle time (e.g., a variation of *Red Light, Green Light)*, with effect sizes that exceeded some prior studies of comprehensive classroom curricula targeting self-regulation (Schmitt et al., 2015). A combination of games and exercise has also demonstrated initial efficacy in promoting preschoolers’ self-regulation (Healey and Halperin, 2015). Developing a game-based intervention that focuses on a single, structured board game can further facilitate delivery of game-based interventions in a manner that minimizes burden and maximizes fidelity and cost-effectiveness. In addition to being relatively low in cost, board games offer an opportunity for scale and equitable reach across sociodemographic groups. Game-based learning has also been shown to optimize participant engagement, compared to traditional cognitive training approaches (Boendermaker et al., 2017). Approaches that are appealing to children can facilitate the repeated practice needed to bolster regulatory skills (Muraven et al., 1999). Further, face-to-face social interactions, which occur during board game play, are beneficial for child development (Corsaro, 2014).

Board games have been used as an inexpensive and effective means to promote traditional academic skills, such as numeracy, among preschoolers from low-income households: After four, 15-min sessions playing a board game involving counting along a number line, preschoolers demonstrated significant improvements in numeracy (Siegler and Ramani, 2008). These findings have been robust across studies in the laboratory and classroom (Ramani and Siegler, 2008; Siegler and Ramani, 2009; Ramani et al., 2012), supporting the use of board games in interventions with preschool children. While no study has used a board game to promote DG specifically, more traditional approaches promoting this and related self-regulation skills have highlighted key facets of successful interventions, including that these skills are more likely to be improved when: (1) they are modeled by others (Corriveau et al., 2016; Haimovitz et al., 2019; Munakata et al., 2020); (2) they are practiced and progressively challenged (Diamond and Lee, 2011); and (3) outcomes are shown to be predictable (Kidd et al., 2013; Michaelson et al., 2013). For example, preschool children were more likely to successfully complete a DG task after watching an adult successfully wait for the delayed reward (Corriveau et al., 2016). In another study, preschoolers’ delay behaviors were affected by the extent to which the experimenter could be trusted: if the experimenter promised to bring attractive art supplies and followed through, preschoolers waited longer in a subsequent DG task, compared to a condition in which preschoolers learned to doubt the reliability of the environment after the experimenter did not deliver the promised supplies (Kidd et al., 2013). These factors were taken into consideration in developing the study game described herein.

The goal of the present study was to test the feasibility and initial efficacy of a new board game designed to promote DG skills via two experimental studies. We hypothesized that preschool children’s likelihood of delaying gratification in the context of the study game would increase across game-play sessions, and that children randomized to play the study game would also increase their DG in a standard lab task compared to controls.

## Methods

### Participants

#### Study 1

Parents and their preschool-aged children were recruited using multiple methods, including advertisements on social media, tables at community events, and flyers in community settings such as childcare centers. Inclusion criteria were: parent ≥18 years old and English speaking and child 4-to-5 years old, English speaking, with no health conditions or allergies precluding participation. There were 221 responses to a screening questionnaire and 191 eligible families. Those not enrolled were later contacted about Study 2 described below. Fifty-six children and one of their parents were recruited into Study 1 and randomized to the Gem Heroes board game (intervention) or control group. DG data were not usable for five of 53 study completers (e.g., due to child distress), leaving 48 families in the analytic sample (*n* = 25 intervention, *n* = 23 control). Sociodemographic characteristics of the sample are shown in Table 1.

**TABLE 1:** Study 1 participant characteristics.

	Mean ± SD or Frequency (%)	
	Randomized families (*n* = 56)^a^	Analytic sample (*n* = 48)
***Child***		
Sex	67.9% boys, 32.1% girls	68.8% boys, 31.3% girls
Age	4.79 ± 0.52 years	4.81 ± 0.55 years
Ethnicity	1.8% Hispanic/Latino (*n* = 55)	2.1% Hispanic/Latino (*n* = 47)
Race	76.4% White, 10.9% Black, 12.7% Multiracial (*n* = 55)	76.6% White, 12.8% Black, 10.6% Multiracial (*n* = 47)
How often child plays board games	14.3% all the time, 73.2% sometimes, 7.1% hardly ever, 5.4% never	16.7% all the time, 68.8% sometimes, 8.3% hardly ever, 6.3% never
***Parent***		
Sex	92.9% female, 7.1% male	93.8% female, 6.3% male
Age	36.0 ± 5.7 years	36.3 ± 5.9 years
Ethnicity	1.8% Hispanic/Latino (*n* = 55)	2.1% Hispanic/Latino (*n* = 47)
Race	87.3% White, 10.9% Black, 1.8% Asian (*n* = 55)	85.1% White, 12.8% Black, 2.1% Asian (*n* = 47)
Education	12.5% HS, 25.0% some college/Associate’s, 19.6% BA/BS, 42.9% graduate degree	8.3% HS, 27.1% some college/Associate’s, 20.8% BA/BS, 43.8% graduate degree
Marital status	73.2% married, 5.4% living with partner, 19.6% never married or divorced/separated, 1.8% other	72.9% married, 4.2% living with partner, 20.8% never married or divorced/separated, 2.1% other
***Household***		
Child eligible for free or reduced-price school meals	60.7% no, 19.6% yes, 19.6% don’t know	60.4% no, 16.7% yes, 22.9% don’t know
Annual household income	7.1% < $14,999, 5.4% $15,000–$24,999, 7.1% $25,000–$34,999, 7.1% $35,000–$49,999, 25.0% $50,000–$74,999, 21.4% $75,000–$99,999, 26.8% > $100,000	6.3% < $14,999, 6.3% $15,000–$24,999, 6.3% $25,000–$34,999, 6.3% $35,000–$49,999, 29.2% $50,000–$74,999, 18.8% $75,000–$99,999, 27.1% > $100,000

*^*a*^Sample sizes for the randomized and analytic samples are 56 and 48. Data shown here correspond to those sample sizes unless otherwise stated (e.g., for race/ethnicity, sample sizes are slightly smaller due to a participant preferring not to answer). 100% of participating parents reported being a primary caregiver of the child. In cases where percentages do not total 100, this is due to rounding. HS, high school; BA/BS, Bachelor’s degree.*

#### Study 2

Parents and children were recruited using similar methods as Study 1, with the addition of postcards mailed to lower-income zip codes of Buffalo, New York. In Study 2, we aimed to repeat the test of our board game intervention in a sample with greater room for improvement in DG. Recruitment, screening, and initial inclusion criteria were similar to Study 1, with 3-to-5-year-old children included. There were 438 interested families, with 329 eligible and 141 visiting the laboratory for further screening. Only children with room to improve their DG (i.e., they did not wait the full time during a baseline Marshmallow Test) were invited to proceed. These participants (*n* = 54) were randomized and continued with remaining study sessions; 50 had post-test DG data and make up the analytic sample (*n* = 22 intervention, *n* = 28 control). Sociodemographics are shown in Table 2. There were no group differences on baseline sociodemographic characteristics in Study 1 or 2 (*p* > 0.10).

**TABLE 2:** Study 2 participant characteristics.

	Mean ± SD or Frequency (%)	
	Randomized families (*n* = 53)^a^	Analytic sample (*n* = 50)
***Child***		
Sex	50.9% boys, 49.1% girls	50.0% boys, 50.0% girls
Age	4.01 ± 0.74 years	4.02 ± 0.76 years
Ethnicity	5.8% Hispanic/Latino (*n* = 52)	4.1% Hispanic/Latino (*n* = 49)
Race	78.4% White, 13.7% Black, 2.0% American Indian, 5.9% Multiracial (*n* = 51)	79.2% White, 14.6% Black, 2.1% American Indian, 4.2% Multiracial (*n* = 48)
How often child plays board games	7.5% all the time, 64.2% sometimes, 9.4% hardly ever, 18.9% never	8.0% all the time, 68.0% sometimes, 6.0% hardly ever, 17.0% never
***Parent***		
Sex	96.2% female, 3.8% male	98.0% female, 2.0% male
Age	35.9 ± 5.7 years	36.0 ± 5.7 years
Ethnicity	5.7% Hispanic/Latino	4.0% Hispanic/Latino
Race	78.4% White, 13.7% Black, 2.0% American Indian, 5.9% Multiracial (*n* = 51)	79.2% White, 14.6% Black, 2.1% American Indian, 4.2% Multiracial (*n* = 48)
Education	5.7% HS, 22.6% some college/Associate’s, 34.0% BA/BS, 37.7% graduate degree	6.0% HS, 22.0% some college/Associate’s, 32.0% BA/BS, 40.0% graduate degree
Marital status	82.7% married, 3.8% living with partner, 13.4% never married or divorced/separated (*n* = 52)	81.6% married, 4.1% living with partner, 14.3% never married or divorced/separated (*n* = 49)
***Household***		
Child eligible for free or reduced-price school meals	54.7% no, 20.8% yes, 24.5% don’t know	56.0% no, 20.0% yes, 24.0% don’t know
Annual household income	5.7% < $14,999, 5.7% $15,000–$24,999, 7.6% $25,000–$34,999, 13.2% $35,000–$49,999, 22.6% $50,000–$74,999, 17.0% $75,000–$99,999, 28.3% > $100,000	4.0% < $14,999, 6.0% $15,000–$24,999, 6.0% $25,000–$34,999, 12.0% $35,000–$49,999, 24.0% $50,000–$74,999, 18.0% $75,000–$99,999, 30.0% > $100,000

*^*a*^Sample sizes for randomized and analytic samples depicted above are 53 and 50.54 participants were randomized and attended game play Session 1, where the surveys were completed, but technological difficulties during survey completion resulted in a sample size of 53. Data shown here correspond to the aforementioned sample sizes unless otherwise stated (e.g., for race/ethnicity, sample sizes are slightly smaller due to prefer not to answer responses). 100% of participating parents reported being a primary caregiver of the child. In cases where percentages do not total 100, this is due to rounding. HS, high school; BA/BS, Bachelor’s degree.*

### Intervention

#### Study 1

The Gem Heroes board game was designed to promote preschoolers’ DG. Different game prototypes were “play-tested” with a separate sample of 10 4-to-5-year-old children, and a finalized version of the game showing initial evidence of efficacy, appeal, and clarity was selected (Supplementary Figure 1). Its premise is: the power has gone out in “Futureville” because a lightning bolt struck the city’s power crystal, breaking it into many sparkly gems. To help restore power to the city, players must collect gems; the winner is the one with the most gems at the end. Children spin a spinner to move along the game board. On certain spaces (action, or “POW,” spaces), players must decide whether they would like a sparkly gem now or a (boring) sidekick that can help them later. Although selecting a gem now might be tempting, since collecting sparkly gems is fun and will help the child win the game, the lesson to be learned is that waiting pays off. At two specific “gates,” each sidekick can be traded for multiple gems, so selecting sidekicks leads to reliable increases in gems in the end. The adult playing the game models choosing sidekicks, with scripted language to scaffold delay behaviors.

Halfway through the study sessions, an additional component is added to the game: children can build the power crystal by arranging collected gems on a power crystal design. This additional aspect of game play was included given evidence that regulatory skills are most likely to be bolstered when challenges increase in complexity over time (Diamond and Lee, 2011). The idea is that, after learning that choosing “boring sidekicks” pays off in the initial weeks of play, the gems will become increasingly tempting when there is a fun new opportunity to engage with them in the short term. Overall, mechanics of the game fit with aforementioned past research supporting greater improvement of DG skills when: (1) they are modeled by others (Corriveau et al., 2016; Haimovitz et al., 2019); (2) they are practiced and progressively challenged (Diamond and Lee, 2011); and (3) outcomes are shown to be predictable (Kidd et al., 2013; Michaelson et al., 2013).

The intent was for the game to be played in groups of three (two children and one adult), with groups of players determined a *priori* and groups kept consistent over time. Research assistants were trained to lead the study game adhering to a script (available from the corresponding author upon request). The researchers also led the playing of Zingo!^®^, a commercially available, bingo-like game, which was selected for the control group because it is a highly rated, age-appropriate game which does not involve taking turns or delaying gratification.

#### Study 2

The games were the same as Study 1, with minor additions to Gem Heroes based on learnings from Study 1 and the literature: (1) a pre-game simulation, (2) the use of psychological distancing, and (3) additional incremental challenges. First, based on observations that children who learned to delay in the context of the game often did so after playing one full round (and seeing the implications of gem/sidekick trades at the finish), we added a brief demonstration before the first game play session, intended to provide this learning experience from the start. Children each received two gems and the adult two sidekicks. Then all players “zoomed” to the finish to simulate what happens at the end of the game, witnessing how those with sidekicks end up accumulating more gems after the trades that are possible at the final gate. This strategy parallels a successful approach in an experimental study by Imuta et al. (2014), in which illustrating the difference between the immediate reward and the delayed reward in a context that emphasizes “cool” rather than “hot” emotional features of the decision bolstered children’s delay skills (here, showing the trade-off in a context that will not yet affect the child’s outcome in “real” game play).

After this simulation, game play began. Children were coached to think of themselves as the superhero game piece they selected to use throughout the game and to envision qualities of this superhero, including patience. They gave their superhero a name (e.g., Strong Girl), and the adult referred to the child as such throughout the game. Previous research has demonstrated that “psychological distancing” by envisioning oneself as a character or hero can aid children in delaying gratification (Karniol et al., 2011) and executive functioning (White and Carlson, 2015). Finally, children only played to the first gate (i.e., half of the board) during each round of game play during the first 2 weeks. Then they “zoomed” to the finish and began trading in their sidekicks to see who won. This modification shortened the DG demands in the initial rounds of game play, supporting the development of these skills in an incremental manner (Diamond and Lee, 2011).

### Study Procedures

All study procedures were approved by the University at Buffalo Institutional Review Board (see Supplementary Figure 2). Parents provided written informed consent, and children provided verbal assent prior to study procedures. Doses of game play were based on prior research, in which four, 15-min game play sessions resulted in improvements in the outcome of interest (numeracy skills) among preschoolers (Siegler and Ramani, 2008).

#### Study 1

Families visited the lab for 4 weeks. Session 1 involved a baseline assessment of DG, followed by 20 min of playing the assigned game with two other individuals (another child and the experimenter, or if only one child was present that day, two study staff played). Sessions 2–4 involved 20 min of game play, with a post-test DG assessment at the end of Session 4.

#### Study 2

Study completers visited the lab for 5 weeks. The baseline assessment of DG determined subsequent eligibility. Those invited to return were randomized and played their assigned game during 4 weekly sessions for 15 min per session, with a post-test DG assessment administered at the end of the last session.

### Measures (Both Studies)

#### Delay of Gratification During the Study Game

Researchers watched video feeds of game play and recorded decisions made by intervention-group children at each “POW” space: i.e., gem or sidekick, the latter of which is consistent with delaying gratification.

#### Delay of Gratification Laboratory Task

Our primary assessment of between-group differences in DG was Mischel’s “Marshmallow Test” (Mischel et al., 1989), which was administered at baseline, prior to any game play, and at post-test after the four game play sessions. This task has been used extensively to assess DG in 3-to-5-year-old children and has demonstrated convergent validity (Duckworth and Kern, 2011). A researcher asked the child which s/he liked better, marshmallows or chocolates, and presented two plates of the preferred treats (2 vs. 6 mini marshmallows or mini chocolate bars) and a bell, telling the child that she needed to leave the room. The child was told that if s/he could wait while the researcher was gone, s/he could have the bigger pile of food. If the child could not wait, s/he could ring the bell and eat the smaller pile. The researcher asked questions to ensure that the child understood the rules, reiterating details as necessary. The wait period was 10 min, which was expected to elicit individual differences (Francis and Susman, 2009). A live video feed was monitored to record seconds the child waited. The researcher stopped the task when the child rang the bell, ate the food, or when 10 min passed. The primary operationalization of this variable was whether wait times increased from baseline to post-test (defined as a change greater vs. less than or equal to 0 s).

#### Feasibility

After the first and final game play sessions, children were asked how much fun it was to play the game and how easy it was to understand, each on a three-point visual face scale.

#### Demographics Parent Survey

Parents self-reported demographics, including child age, sex, race/ethnicity, and free- or reduced-price school meal eligibility, and parent age, education, and annual household income, using an electronic tablet. Parents also reported how often their child typically plays board games using questions adapted from prior board game research (Siegler and Ramani, 2008).

### Data Analysis

#### Sample Size Calculations

Initial sample size estimates used effect sizes from an experiment in which preschoolers’ (median = 4.5 years) attention to rewards was manipulated during the Marshmallow Test (Mischel and Ebbesen, 1970). Mean waiting times in “no rewards” and “delayed rewards” conditions were used to approximate expected differences between groups allocating differing amounts of attention to rewards due to the intervention (6.4 min). This led to an estimate of 30 children needed to detect differences in wait times at 80% power and alpha = 0.05, which we increased to 56, given unknown factors when conducting a first investigation in a new area (e.g., potential intraclass correlations among groups of children playing together; potential unexpected events such as unusable data; attrition). We used observed effect sizes from Study 1 to confirm that a similar sample size goal was appropriate for Study 2.

#### Study 1

We explored intraclass correlations to assess whether groupings in which children played their game should be included in analyses. Intraclass correlations were small (<0.02), so we proceeded with the following analyses. To examine changes in DG during game play, we conducted a generalized linear model with a binomial distribution, examining whether intervention-group children chose the short-term (gem) or longer-term reward (sidekick) at each “POW” space. This model included fixed effects of session (1–4) and decision number (decision at the 1st “POW” space child landed on, 2nd, etc.) and a random effect for subject. We included the first 8 decision points during the first round of the game of each session. Including more decision points led to challenges with model convergence, given that there is an element of chance determining how many “POW” spaces a participant experiences. We examined overall fixed effects of decision and session number, and in cases in which one or both of these was significant, planned to examine least squares means to shed light on the nature of the change: e.g., if there was a significant effect of session, we planned to examine likelihood of waiting for the long-term reward at Session 1 vs. each subsequent time point until a significant difference emerged, then testing for further changes from that time point. We also planned to examine the overall change in DG from Session 1 to 4.

Next, we used logistic regression to examine effects of study group on Marshmallow Test wait times, specifically whether wait times increased from baseline to post-test. We incorporated covariates implicated by the DG literature (child age, sex, race, and free- or reduced-price meal eligibility, as well as household income and parent education; Evans and English, 2002; Silverman, 2003; Hongwanishkul et al., 2005) and used backward deletion, retaining only covariates that were at least trend-level predictors of DG change (*p* < 0.10). Race, school meal eligibility, and sex were operationalized as categorical variables, child age was continuous, and values were assigned to ordinal education and income variables to allow them to be considered as continuous as well (e.g., translating each educational category to a number of years of education).

We repeated this main logistic regression model with baseline wait times as an additional predictor, dichotomized at 20 s, based on prior research (Watts et al., 2018), and explored potential moderation by aforementioned covariates. We supplemented these analyses with: (1) a generalized linear model operationalizing the Marshmallow Test outcome as wait times that either increased, decreased, or stayed the same; (2) a simple chi-square analysis of the aforementioned dichotomous outcome; and (3) a non-parametric Wilcoxon test examining group effects on the (continuous) difference in DG from baseline to post-test. All of these analyses were suitable for the non-normal distributions of wait times. It is not unusual for the distribution of this DG variable to violate normality assumptions and for it to be examined accordingly (e.g., Francis and Susman, 2009; Watts et al., 2018). Descriptive statistics were also conducted, including: means and standard deviations, medians and ranges, or frequencies of key sociodemographic and outcome variables; bivariate relationships between sociodemographic characteristics and DG variables; and frequencies describing how often the entire time allotted for game play was completed, how often intervention-group children completed a first and second round of game play within a session, number of “POW” spaces intervention-group children landed on during game play, and children’s perspectives on their assigned game.

#### Study 2

Analyses were the same as in Study 1, with two exceptions: when examining changes in DG in the context of game play, we incorporated 6 decision time points into the generalized linear models to avoid aforementioned issues with sparseness. When examining effects of the study game on wait times during the Marshmallow Test, we were unable to run the supplemental model assigning wait times to three categories, as in this study, no child’s wait times remained the same from baseline to post-test.

## Results

Descriptive statistics for key DG variables and bivariate relationships between sociodemographic characteristics and these variables are shown in Supplementary Table 1.

### Study 1

#### Delay of Gratification During Game Play

Most children completed all 20 min of game play (range across the four sessions = 91.7–100.0%). In the intervention group, a first round of game play was almost always completed (96.0–100%), whereas completion of a second round within a 20-min session was rare (4.0–12.0%). All intervention-group participants had the chance to land on at least two POW spaces during each of the four game play sessions, with the majority of the participants landing on five or more (Supplementary Table 2).

There was a significant main effect of session in predicting the likelihood of intervention-group children waiting for the long-term reward (i.e., choosing sidekicks) during game play (*X*^2^ = 9.14, *p* < 0.05). There was no main effect of decision number (*p* = 0.58). Comparisons of least squares means showed a significant increase in the likelihood of delaying gratification from Session 1 to 2 (OR = 3.16, *p* < 0.01), with no further significant changes from Session 2 to 3 or 4, and a significant change from Session 1 to 4 overall (OR = 5.36, *p* < 0.001).

#### Delay of Gratification During the Marshmallow Test

Univariate statistics showed that Marshmallow Test wait times increased by an average of 24.64 ± 200.75 s (Median = 0, Range = −465–564) in the intervention group, while the control group’s wait times decreased by 18.48 ± 186.54 s (Median = 0, Range = −476–572). The intervention group was 7.12 times more likely to increase wait times from baseline to post-test versus controls (*p* = 0.048; 95% CI: 1.02, 49.69), with child age and parent education retained in the primary logistic regression model after backward deletion. Older children and children with parents of a higher education level tended to be less likely to increase their DG (OR = 0.88, *p* = 0.079; OR = 0.65, *p* = 0.052, respectively). After adjusting for baseline wait times, results were similar, but the group effect became a trend (*p* = 0.06). There was no evidence of moderation by demographics.

Results were similar in supplementary chi-square and Wilcoxon (*p* = 0.09) analyses and when operationalizing change in wait times in three categories. Compared to the control group, the intervention group tended to be more likely to increase their wait times vs. wait times staying the same (*p* = 0.05); they were also more likely to increase their wait times vs. wait times decreasing (*p* = 0.07). There were no study group differences in the likelihood of wait times staying the same vs. decreasing. Many children in this sample were already waiting the full 10 min during the baseline DG assessment (*n* = 32, 66.7%). When examining only those who had room to improve their DG at baseline, intervention group wait times increased by an average of 120.11 ± 282.04 s (Median = 11, Range = −394−564), while the control group’s wait times increased by 7.29 ± 301.78 s (Median = −7.0, Range = −277−572).

#### Feasibility

After the first session of game play, intervention-group children’s perspectives on the study game were positive, including 92.0% of children reporting that the game was very fun and 75% reporting that it was easy to understand. Similarly, in the control group, the majority of children rated their game as very fun to play (95.7%) and easy to understand (87.0%). By the final session of game play, 72.0% of the intervention group rated the study game as very fun, and 84.0% thought it was easy to understand. In the control group these numbers were 91.3% and 78.3%, respectively (Table 3).

**TABLE 3:** Child-reported feasibility and acceptability data (%).

	Intervention Session 1	Intervention Session 4	Control Session 1	Control Session 4
***Study 1 (intervention group n = 25; control n = 23)***				
*How fun was game?*				
Very fun	92.0	72.0	95.7	91.3
Sort of fun	8.0	20.0	0.0	8.7
Not fun	0.0	8.0	4.4	0.0

*How easy was game to understand?*				
Very easy	76.0	84.0	87.0	78.3
Neutral	16.0	8.0	4.4	4.4
Too hard	8.0	8.0	8.7	17.4

***Study 2 (intervention group n = 22; control = 28)***				

*How fun was game?*				
Very fun	81.0^a^	86.4	85.2^b^	85.7
Sort of fun	4.8^a^	13.6	11.1^b^	3.6
Not fun	14.3^a^	0.0	3.7^b^	10.7

*How easy was game to understand?*				
Very easy	81.0^a^	81.8	89.3	82.1
Neutral	9.5^a^	18.2	3.6	10.7
Too hard	9.5^a^	0.0	7.1	7.1

*Sample sizes are as listed except in the following instances in which not all children chose to respond to the question: ^*a*^*n* = 21, ^*b*^*n* = 27. In cases where percentages do not total 100, this is due to rounding.*

### Study 2

#### Delay of Gratification During Game Play

Most children completed all 15 min of game play (range across sessions = 96.0–100%). In the intervention group, a first round of game play was usually completed (72.7–95.5%). Completion of a second round of game play occurred about half of the time during the first two sessions (45.4–57.1%). Once children progressed to full board game play in Sessions 3 and 4, the 15-min session was not long enough to complete a second round of play (0.0%). Across Study 2’s sessions, at least 72% of participants landed on at least two POW spaces, and the majority landed on three or more (Supplementary Table 2).

There was a significant main effect of session in predicting the likelihood of intervention-group children waiting for the long-term reward during game play [*X*^2^(3) = 8.2, *p* < 0.05] and no main effect of decision number (*p* = 0.20). There was a significant increase in the likelihood of delaying gratification from Session 1 to 2 (OR = 3.61, *p* < 0.01), with no further changes from Session 2 to 3 or 4, and a significant change from Session 1 to 4 overall (OR = 3.20, *p* < 0.05). The pattern of these results was consistent between Study 1 and Study 2, with a greater magnitude of change overall in Study 1. Complete data on decisions made during game play are shown in Supplementary Table 2.

#### Delay of Gratification During the Marshmallow Test

**FIGURE 1:**
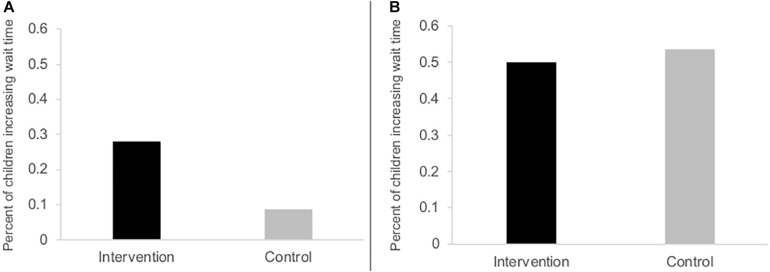
Overall change in delay of gratification by study group in Study 1 **(A)** and Study 2 **(B)**. These histograms depict the percentage of participants in the analytic sample (*n* = 48 and *n* = 50, respectively) who increased their delay of gratification, as indicated via their wait times during the Marshmallow Test at baseline and post-test. In Study 1, the study groups differed (*p* < 0.05 in logistic regression analysis), whereas the overall study group difference in Study 2 was not significant. At baseline, all participants in Study 2 had room to improve their delay of gratification (i.e., waited less than 10 min during their baseline assessment), a design modification added after Study 1.

Univariate statistics showed that Marshmallow Test wait times increased by an average of 85.64 ± 249.87 s (Median = 0.50, Range = −444−592) in the intervention group, while the control group’s wait times increased by 100.61 ± 239.69 s (Median = 8, Range = −263−590). There was no significant effect of study group on changes in DG during the Marshmallow Test (*p* = 0.27), with child eligibility for free- or reduced-price meals and family income retained after backward deletion. Children of parents indicating eligibility for free- or reduced-price meal eligibility or a lack of knowledge about eligibility were both more likely to increase their DG than children of parents indicating ineligibility (OR = 29.15, *p* = 0.006, and OR = 10.72, *p* = 0.016, respectively). In the context of this model adjusted for school meal eligibility, there was also a trend such that higher income was associated with a greater likelihood of increasing DG (*p* = 0.058). Non-significant overall study group effects remained when adjusting this model for baseline wait times and in chi-square and Wilcoxon analyses (*p* > 0.31), and there was no evidence of moderation by demographics. Given Study 2’s inclusion criteria, all participants had a wait time <600 s at baseline (Supplementary Table 1). Half (52.0%) increased their wait times from baseline to post-test. Figure 1 shows this frequency by group for both studies.

#### Feasibility

After the first session of game play, 81.0% of the intervention group thought the study game was very fun, and 81.0% thought the game was easy to understand. Similarly, in the control group, the majority of children rated their game as very fun to play (85.2%) and easy to understand (89.3%). By the final session of game play, 86.4% of the intervention group rated the study game as very fun, and 81.8% thought it was easy to understand. In the control group these numbers were 85.7 and 82.1%, respectively.

## Discussion

Results from this pair of studies support continued investigation of the Gem Heroes board game. Most children rated the study game favorably, supporting feasibility, and in both studies, children’s delay behaviors increased over time in the context of the study game. Further, there was some evidence consistent with the hypothesis that playing this board game would increase children’s DG in a separate context (the Marshmallow Test), although the nature of these results differed by study. In Study 1, intervention-group children were more likely to increase their wait times from baseline to post-test relative to controls. In Study 2, there was no overall effect of the study game on changes in Marshmallow Test wait times.

The consistent changes in delay behaviors observed during Gem Heroes game play support the hypothesis that playing this game can help increase DG through the experience of seeing delay behaviors pay off. There was a significant increase in selection of the delayed reward from the first to the final session of game play, with the increase emerging between the first and second sessions. This pattern was similar between the two studies, with a smaller magnitude of change in Study 2.

While changes in the context of the game were generally consistent across studies, results differed when considering impacts of the game on wait times during the Marshmallow Test. The changes in inclusion criteria and game play between Study 1 and Study 2 should be considered in interpreting the results. For example, the game play sessions changed from 20 min in duration to 15 min from Study 1 to Study 2. While the latter is still consistent with prior efficacious board game research (Siegler and Ramani, 2008), the 20-min sessions may have afforded greater learning in the context of game play. During Sessions 3–4, when a round of game play was consistently defined as use of the full game board, a significantly greater number of rounds of game play were completed in Study 1 vs. Study 2. Analyses of children’s decision-making suggest that successive rounds of game play present the opportunity to learn the value of waiting for the delayed reward. Other changes made between Study 1 and Study 2 included minor changes to the game itself based on emerging evidence on promoting DG skills. It is not possible to determine exactly which of these changes (if any) are responsible for differences in findings across the two studies. Factorial designs could be used to compare different versions of the game in the future to provide further insights on its potential to impact DG skills. It is also important for future randomized studies of DG interventions to elucidate the extent to which modifying DG affects outcomes like achievement and weight status across different sociodemographic groups.

Analyses of bivariate relationships between sociodemographics and DG in the context of the present research revealed some expected findings, such as links between a lower education level and lower DG in Study 1 and links between free- and reduced-price meal eligibility and greater increases in DG in Study 2. However, the nature of these relationships varied across the two studies, and relationships were not confirmed in the case of every sociodemographic variable. Given the relatively homogeneous nature of the present samples, future research could explore the potential of the study game to impact DG in lower-SES samples. We recently conducted some initial pilot research with children enrolled in Head Start (*n* = 10), which showed feasibility of intervention implementation and apparent similarities in DG change in the context of game play in that setting (e.g., 10% of children selected the delayed reward at their first “POW” space the first time they played the game and 40% the second time). Literature on DG suggests that children from lower-SES households are less likely to delay (Evans and English, 2002), and there is evidence supporting the idea that these differences reflect adaptive responses to unpredictable environments, rather than deficits (Sturge-Apple et al., 2016). Future tests of this or other interventions aiming to promote DG should consider whether interventions are contextually appropriate.

When asked about how much fun the study game was and how easy it was to play, the majority of children in the present sample responded favorably, supporting feasibility. We did notice that enthusiasm for the game appeared to decrease slightly from baseline to post-test in Study 1 while this did not appear to happen as much in Study 2. This may have been due to the incremental changes in game play in Study 2, which were designed to help build the child’s understanding of the game in an incremental manner; this also meant that individual rounds of the game were not the same – or quite as long – across all four game play sessions. Future research could clarify the extent to which such factors influence children’s impressions of the game, but it is encouraging that the majority of children responded positively across the study period during both studies.

Limitations of these studies include the relatively homogeneous samples and the inability to conclude with certainty which of the changes between Study 1 and Study 2 are responsible for the differences in results, if any. Further, 32 of the 48 of the children in Study 1 waited the full time during the baseline Marshmallow Test, constraining the possibility for change; 18 of the 48 children showed changes in wait times from baseline to post-test in this study. We also have limited information about the ways children were interpreting the provision of the different rewards. For example, were the gems rewarding in the moment because they were attractive, sparkly, and fun to interact with, and/or because they were linked with the anticipation of winning the game? The gems may not have afforded the same short-term rewards as the immediate consumption of the marshmallow during the Marshmallow Test. Future research could seek to better understand the nuances of children’s decision-making processes during this game. Strengths of this study include the randomized design and the evaluation of a simple, low-cost intervention with the potential to promote DG among preschool children. The experience of practicing DG during game play may be particularly important and impactful among children who are growing up in contexts that are less predictable. Future studies could continue to refine and test the study game to elucidate its potential effects among different sociodemographic groups. If future studies support the efficacy of this game, it may also be considered as part of broader efforts targeting interrelated sociodemographic and behavioral factors (Falk et al., 2020; Michaelson and Munakata, 2020; Watts and Duncan, 2020).

## Data Availability Statement

The raw data supporting the conclusions of this article will be made available by the authors upon reasonable request and following any necessary Institutional Review Board approval, without undue reservation.

## Ethics Statement

The studies involving human participants were reviewed and approved by the University at Buffalo Institutional Review Board. Written informed consent to participate in this study was provided by the participants’ legal guardian/next of kin.

## Author Contributions

SA-F designed and led the study, conducted analyses, and drafted the manuscript. AS coordinated the study, collected the data, and contributed to data management. DC and DP contributed to game design. LE and MF contributed to study design and data interpretation. ST contributed to data collection and data management. KR contributed to data collection. All authors provided critical feedback on the manuscript.

## Conflict of Interest

The authors declare that the research was conducted in the absence of any commercial or financial relationships that could be construed as a potential conflict of interest.

## Supplementary Material

Supplementary Figure 1 Gem Heroes board game. This image shows the full intervention board game set up.

Supplementary Figure 2 Study procedures. The study procedures for Study 1 are shown in Panel A, and Study 2’s procedures are in Panel B.
