# Supplementary figures and images for: Evaluating a Board Game Designed to Promote Young Children’s Delay of Gratification
Source: Front Psychol. 2020 Nov 11;11:581025. doi: 10.3389/fpsyg.2020.581025 (PMC7686572; doi:10.3389/fpsyg.2020.581025)

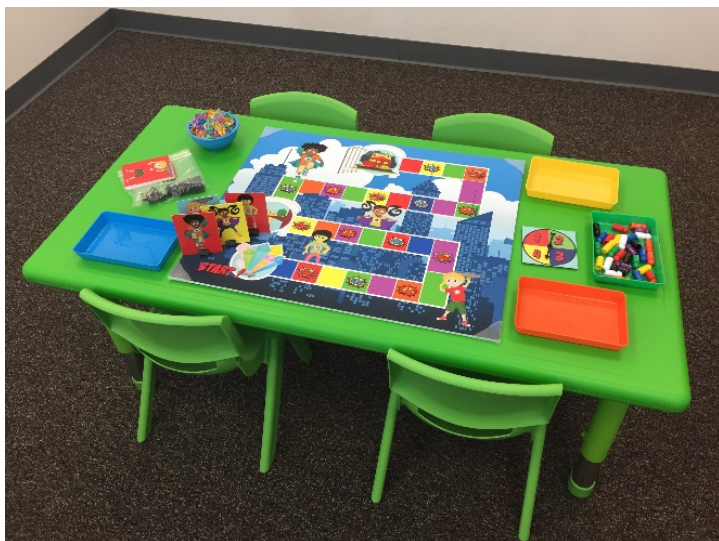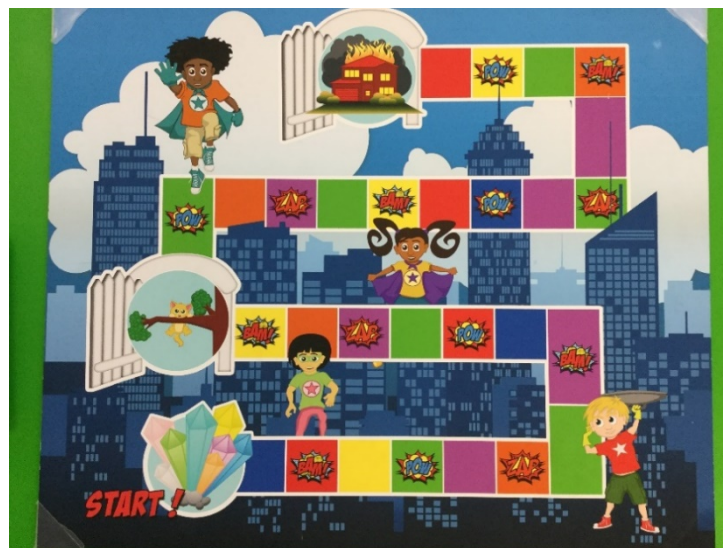

SUPPLEMENTAL FIGURE 1

Supplement: Supplementary Figure 1 — Gem Heroes board game. This image shows the full intervention board game set up. [file Image_1.pdf]

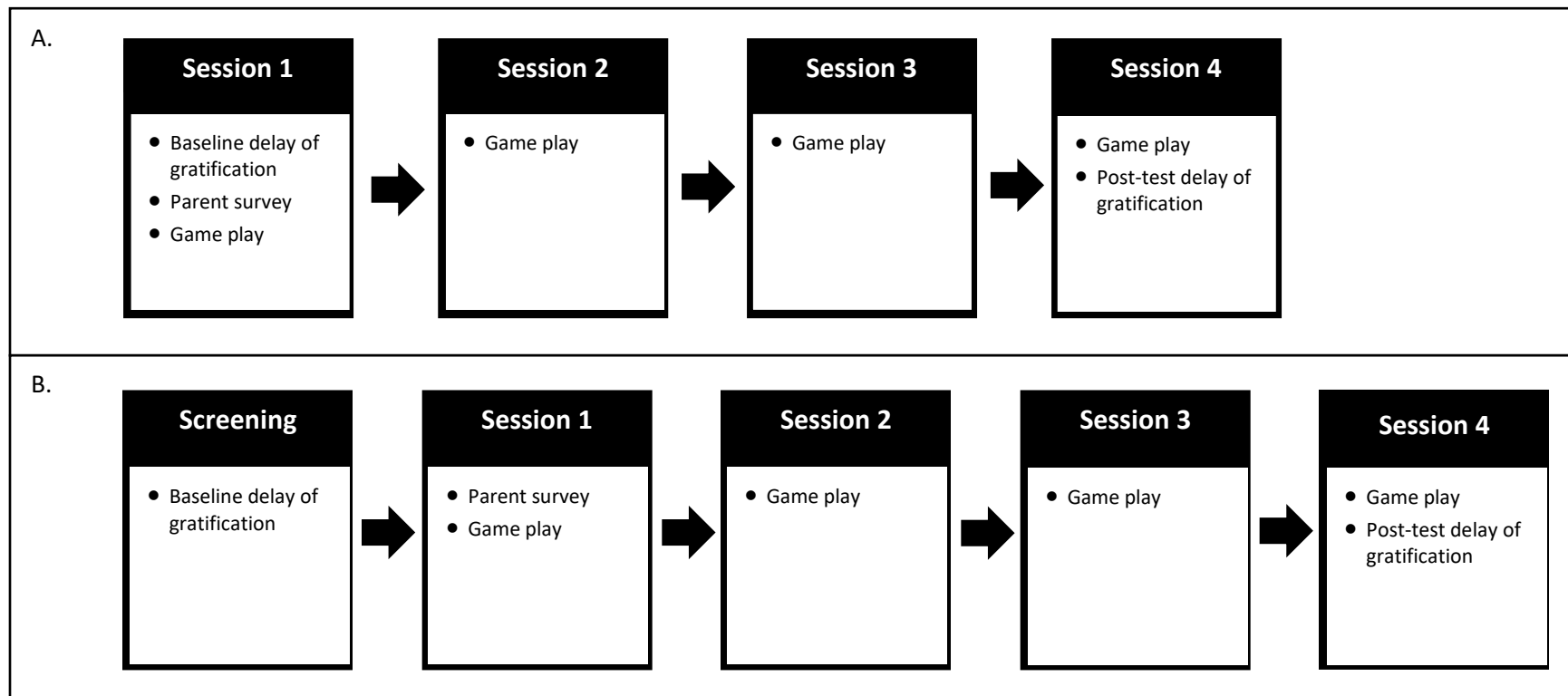

**SUPPLEMENTAL FIGURE 2**

Supplement: Supplementary Figure 2 — Study procedures. The study procedures for Study 1 are shown in Panel A, and Study 2’s procedures are in Panel B. [file Image_2.pdf]
