# Supplementary material for: Evaluating a Board Game Designed to Promote Young Children’s Delay of Gratification
Source: Front Psychol. 2020 Nov 11;11:581025. doi: 10.3389/fpsyg.2020.581025 (PMC7686572; doi:10.3389/fpsyg.2020.581025)
Supplement: Supplementary file 3 [file Table_1.pdf]

Supplemental Table 1

Changes in Delay of Gratification[1] from Baseline to Post-test: Descriptive Statistics and Bivariate Relationships

	Study 1 (n=48)			*Study 2 (n=50)*		
	Baseline wait time (continuous)	Change in wait time (dichotomous)	Change in wait time (continuous)	Baseline wait time (continuous)	Change in wait time (dichotomous)	Change in wait time (continuous)
Descriptive Statistics						
Median (Range) or %	600.0 (0-600s)	18.8% increased	0.0 (-476-572)	26.0 (3-595s)	52.0% increased	3.0 (-444-592)
Bivariate Relationships with Sociodemographics[2]						
Child						
Sex	r=-0.066	X^2=0.42	r=-0.026	r=0.0042	X^2=0.00	r=0.042
Age	**r=0.29***	r=-0.21	r=-0.095	**r=0.30***	r=-0.049	r=0.039
Race	X^2=1.46	X^2=1.43	X^2=0.95	X^2=2.92	X^2=2.83	X^2=5.42
Parent						
Age	r=0.16	r=-0.041	r=0.10	r=0.13	r=-0.084	r=-0.22
Race	X^2=0.51	X^2=0.28	F=0.021	X^2=2.92	X^2=2.83	X^2=5.42

Education	**r=0.29***	**r=-0.29***	r=-0.083	r=0.070	r=-0.092	r=-0.21
Household						
Free or reduced-price meal eligibility	**X^2=8.66*[3]**	**X^2=6.70*[3]**	X^2=2.49	X^2=2.09	**X^2=7.15*[4]**	**X^2=10.38**[4]**
Income	r=0.17	r=-0.20	r=-0.13	r=0.24#	r=-0.042	r=-0.16

Notes: Some demographic variables were not included due to low variability (e.g., ethnicity, parent sex) as shown in Tables 1 & 2.
#p<.10, *p<.05, **p<.01

[1] Assessed via the Marshmallow Test, possible range = -600 – 600 sec

[2] To test relationships between continuous variables (or one continuous and one dichotomous), Spearman correlations were conducted. In the case of categorical demographic variables, Kruskal-Wallis (continuous outcome) or chi-square (categorical outcome) tests were conducted, each of which yields a chi-square statistic.

[3] These analyses revealed less waiting at baseline and a less likely increase in wait times if the parent indicated that they did not know if the child was eligible for free or reduced school meals (vs. parents who said yes or no).

[4] These analyses revealed less likely increases in waiting if the parent indicated that the child was not eligible for free or reduced price school meals (vs. parents who said the child was eligible or did not know).
