# Supplementary material for: Evaluating a Board Game Designed to Promote Young Children’s Delay of Gratification
Source: Front Psychol. 2020 Nov 11;11:581025. doi: 10.3389/fpsyg.2020.581025 (PMC7686572; doi:10.3389/fpsyg.2020.581025)
Supplement: Supplementary file 4 [file Table_2.pdf]

**Supplemental Table 2**

*Participants' Decisions at POW Spaces during Gem Heroes Game Play by Session (S) and Round (R)*

| | **Number of children (%) choosing delayed reward (sidekick) at each POW space** | | | | | | | |
|---|---|---|---|---|---|---|---|---|
| | Decision 1 | Decision 2 | Decision 3 | Decision 4 | Decision 5 | Decision 6 | Decision 7 | Decision 8 |
| *Study 1 (n=25 in intervention group)* | | | | | | | | |
| S1, R1 | 9/25 (36.0) | 7/25 (28.0) | 6/22 (27.3) | 7/21 (33.3) | 4/14 (28.6) | 3/7 (42.9) | 0/5 (0.9) | 0/1 (0.0) |
| S1, R2 | **13/16 (82.3)** | **9/15 (60.0)** | 8/10 (80.0) | 7/7 (100.0) | 2/3 (66.7) | -- | -- | -- |
| S2, R1 | **17/25 (68.0)** | **16/25 (64.0)** | **15/24 (62.5)** | 10/21 (47.6) | 6/14 (42.9) | 3/7 (42.9) | 3/5 (60.0) | 0/1 (0.0) |
| S2, R2 | **11/19 (57.9)** | 8/11 (72.7) | 8/9 (88.9%) | 5/6 (83.3) | 4/4 (100.0) | 1/1 (100.0) | 1/1 (100.0) | 1/1 (100.0) |
| S3, R1 | 12/25 (48.0) | 9/24 (37.5) | 11/23 (47.8) | **13/21 (61.9)** | 8/17 (47.1) | **8/14 (57.1)** | 6/10 (60.0) | 2/5 (40.0) |
| S3, R2 | **12/14 (85.7)** | 9/10 (90.0) | 5/5 (100.0) | 3/3 (100.0) | -- | -- | -- | -- |
| S4, R1 | **16/25 (64.0)** | **19/25 (76.0)** | **14/25 (56.0)** | **16/22 (72.7)** | **11/15 (73.3)** | 7/9 (77.8) | 5/6 (83.3) | 1/1 (100.0) |
| S4, R2 | **12/15 (80.0)** | 9/10 (90.0) | 6/7 (85.7) | 4/4 (100.0) | 1/1 (100.0) | -- | -- | -- |
| *Study 2 (n=22 in intervention group)* | | | | | | | | |
| S1, R1 | 4/22 (18.2) | 9/22 (40.9) | 5/13 (38.5) | 0/1 (0.0) | -- | -- | -- | -- |
| S1, R2 | 10/20 (50.0) | **10/16 (62.5)** | **7/12 (58.3)** | 1/4 (25.0) | -- | -- | -- | -- |
| S2, R1 | **12/21 (57.1)** | **12/15 (80.0)** | **6/11 (54.6)** | 3/7 (42.9) | 1/3 (33.3) | -- | -- | -- |
| S2, R2 | **11/19 (57.9)** | **13/19 (68.4)** | 7/10 (70.0) | 1/3 (33.3) | 1/2 (50.0) | -- | -- | -- |
| S3, R1 | **16/22 (72.7)** | **13/21 (61.9)** | **12/20 (60.0)** | **10/16 (62.5)** | 5/8 (62.5) | 1/4 (25.0) | -- | -- |
| S3, R2 | 2/7 (28.6) | 4/5 (80.0) | 1/2 (50.0) | 0/1 (0.0) | 0/1 (0.0) | -- | -- | -- |
| S4, R1 | **12/22 (54.6)** | **13/22 (59.1)** | **12/20 (60.0)** | 6/14 (42.9) | 5/8 (62.5) | 2/5 (40.0) | 0/1 (0.0) | -- |
| S4, R2 | 6/11 (54.6) | 4/6 (66.7) | 0/1 (0.0) | 1/1 (100.0) | 0/1 (0.0) | -- | -- | -- |

Notes: These cells depict the number and percentage of children selecting the delayed reward (a sidekick) during each decision point (POW space) of game play. Due to the element of chance when using a spinner, there is some random variability in the number of POW spaces experienced by each child. As such, the denominator of each cell varies (i.e. depicting that most children have the chance to land on at least a few POW spaces per round, but that landing on, for example, 5 or 6 POW spaces is less common). The percentages indicate how often a sidekick is chosen among those who have landed on a given POW space. Cells are bolded when the majority of children chose the delayed reward and at least half of the sample is included (i.e. ≥50% of the sample made it to the POW space in question) in an effort to illustrate instances in which substantial numbers of participants chose the delayed reward.

In Study 1 game play sessions were 20 minutes. In Study 2 they were 15 minutes. In both studies, Session 3 is where tray liners were added to introduce an additional challenge. In Study 2, Session 3 also brings the transition from half to full board game play. This transition results in children experiencing a greater number of POW spaces in Round 1 of game play (vs. Sessions 1 and 2), combined with a lower likelihood of progressing on to a second round within a session. Future research could examine impacts of other combinations of these factors, such as the 20 minutes of game play from Study 1 combined with the transitions from half to full boards in Study 2.

Abbreviations: S=Session (i.e. 1st, 2nd, 3rd, or 4th week of game play). R=Round (i.e. whether it was the 1st or 2nd round of game play within a session).
